# Supplementary material for: Diabetes impairs wound healing by Dnmt1-dependent dysregulation of hematopoietic stem cells differentiation towards macrophages
Source: Nat Commun. 2018 Jan 2;9:33. doi: 10.1038/s41467-017-02425-z (PMC5750226; doi:10.1038/s41467-017-02425-z)
Supplement: Supplementary file 1 — Supplementary Information [file 41467_2017_2425_MOESM1_ESM.pdf]

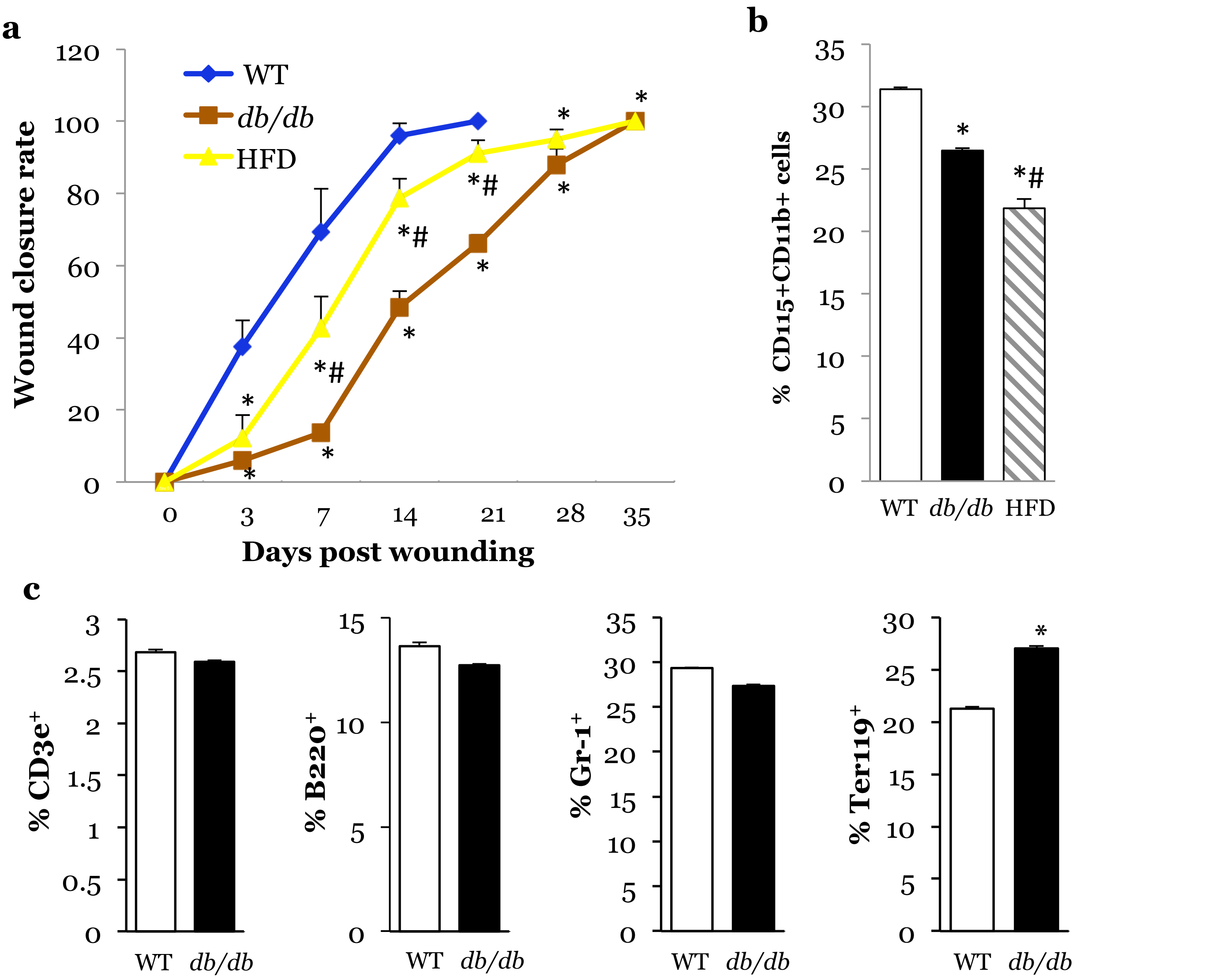


**Supplementary Figure 1. Delayed wound healing and decreased monocytes/ macrophages in high fat diet (HFD) type 2 diabetic mice**. (a) Wound closure rate measurement (n=8.*, p<.05 vs WT; #, p<.05 vs. *db/db*). (b)Flow cytometry analysis of monocytes concentration in the bone marrow in HFD type 2 diabetic mice. (n=6. *, p<.05 vs. WT; #,p<.05 vs. *db/db*). (c ) Flow cytometry analysis of cell lineages in the bone marrow of WT and db/db Type 2 diabetic mice (n=6. *, p<.05 vs. WT). Results are expressed as means ± SEM. One way ANOVA was used for **a** and **b**; two-tailed unpaired student's t-test for **c**.

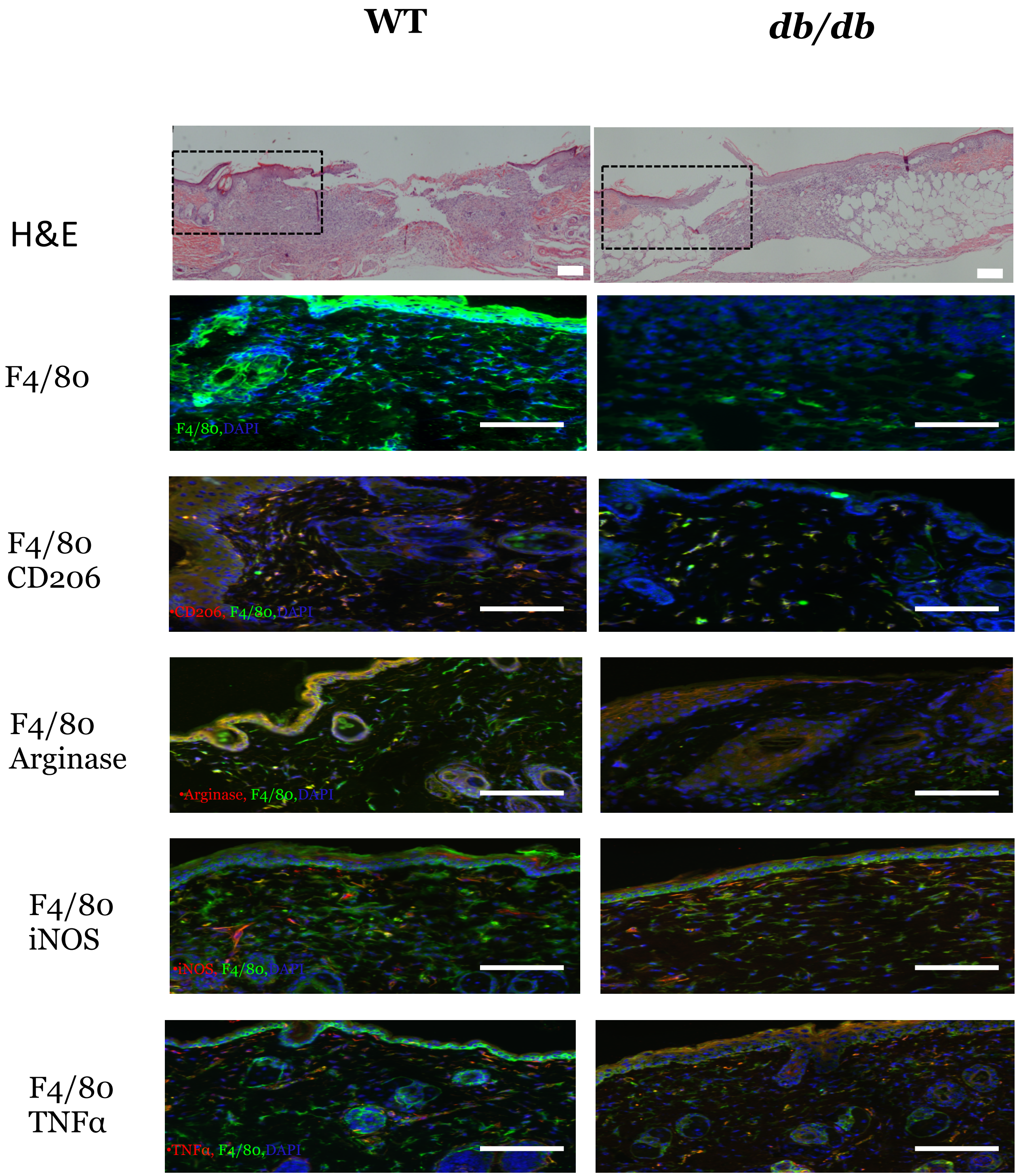


**Supplementary Figure 2. Histological and Immunohistochemical staining of day 7 wound from WT and *db/db* mice.** Marked area in H&E images are the locations for macrophage staining images. Magnification x40 in H&E staining and Magnification x200 in macrophage staining. Scale bar 100µm.

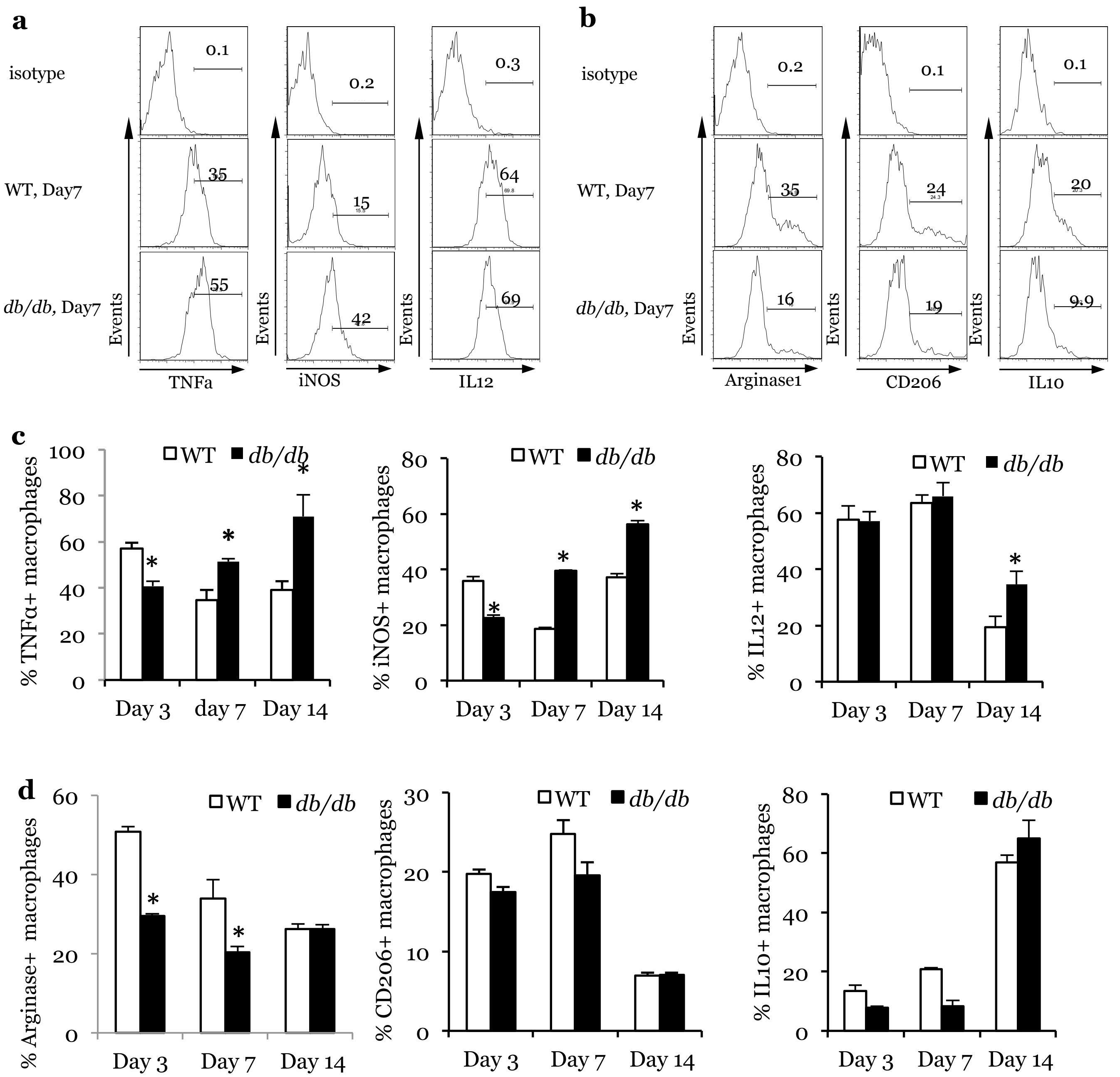


**Supplementary Figure 3. Quantification of M1/M2 macrophage concentration in wounds** (a) Schematic of flow cytometry gating of M1 macrophages. (b) Schematic of flow cytometry gating of M2 macrophages. (c ) Quantification of M1 macrophage concentrations in wounds (n=6, *, P<.05 vs WT). (d) Quantification of M2 macrophage concentrations in wounds (n=6, *, P<.05 vs WT). Results are expressed as means ± SEM. Two-tailed unpaired student's t-test was used for **c** and **d**.

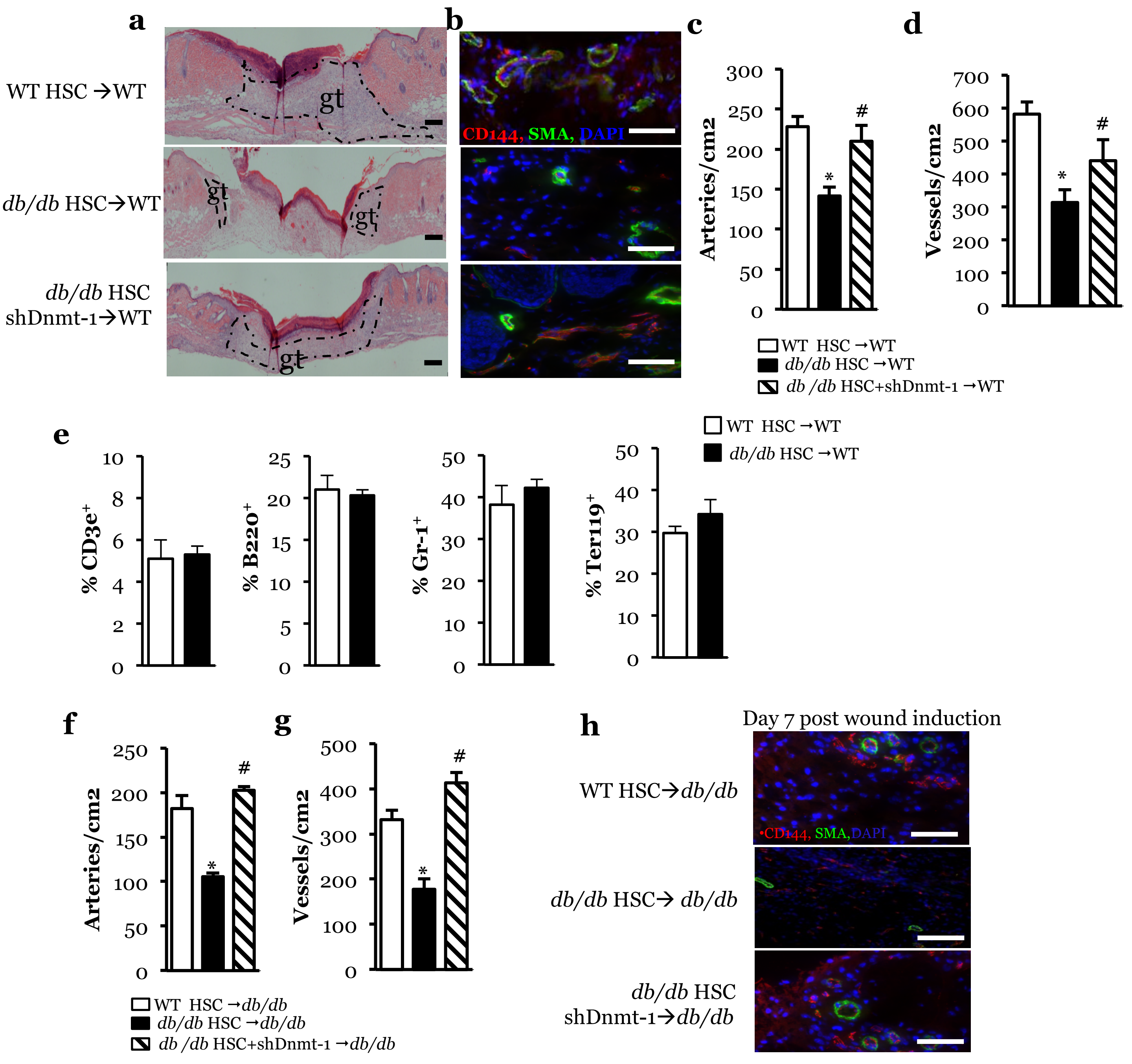


**Supplementary Figure 4. Quantification of vascularization and cell lineages in chimeric mice.** (a) Representative H&E staining images on post wound Day 7 skin (scale bar, 100μm). gt: granulation tissue. (b-d). Quantification of vascularization in day 7 wound in WT recipient mice by CD144 and αSMA staining. Quantification of arteries by CD144 and SMC double positive staining. Quantification of vessels by CD144. Magnification x200. scale bar, 100μm (n=4, *, p<.05 vs WT HSC → WT, #, p<.05 vs *db/db* HSC → WT). (e) Cell lineage analysis by flow cytometry of WT recipient mice. (f-g). Quantification of vascularization in day 7 wound in *db/db* recipient mice by CD144 and αSMA staining. Magnification x200. (n=4, *, p<.05 vs WT HSC → db/db, #, p<.05 vs *db/db* HSC → *db/db*). scale bar, 100μm. Results are expressed as means ± SEM. One way ANOVA was used for **c,d,f** and **g**; two-tailed unpaired student's t-test for **e**.

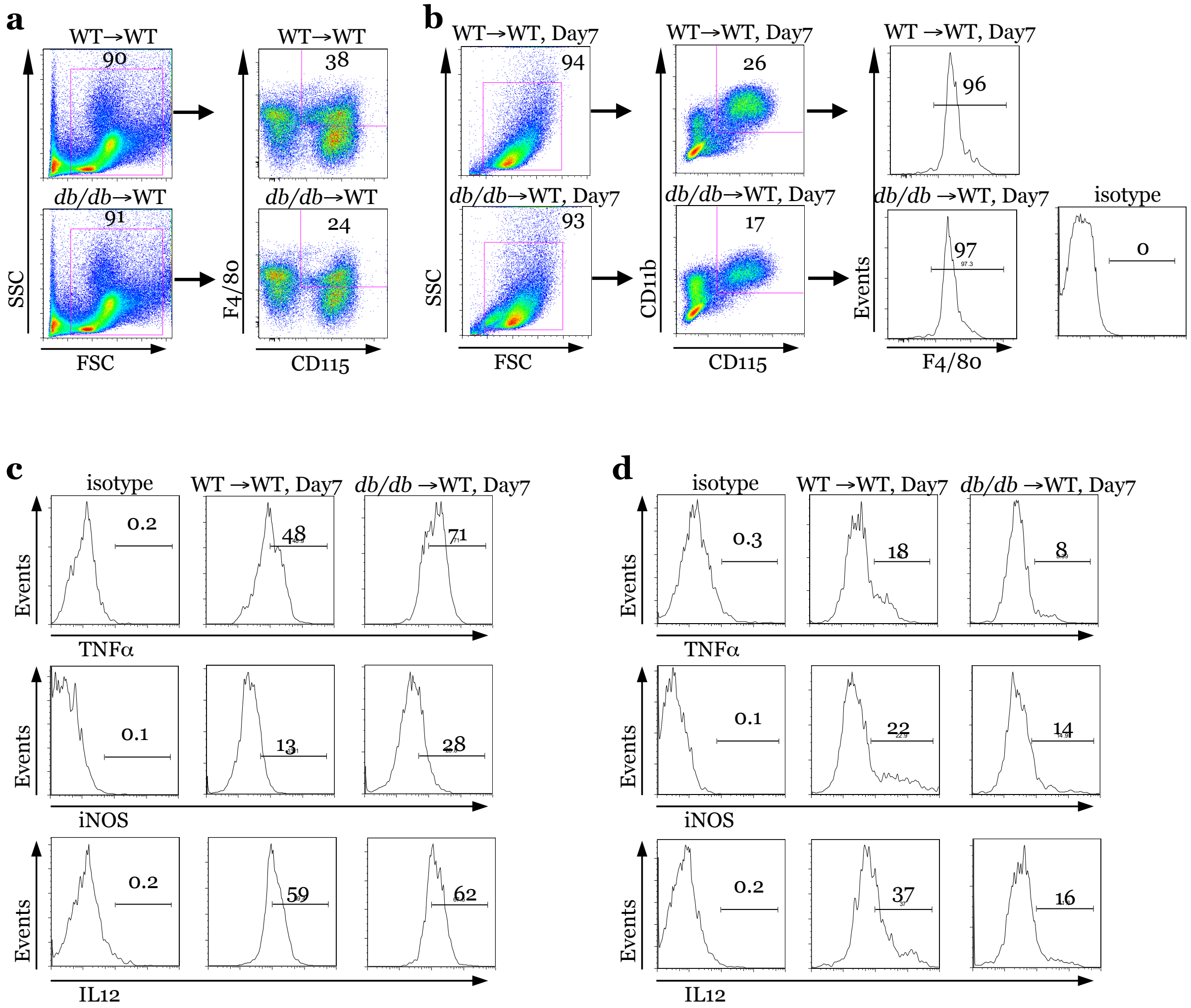


**Supplementary Figure 5. Schematic of flow cytometry gating in WT recipient mice.** Flow cytometry gating of the (a) monocyte population in the bone marrow in WT recipient mice; (b) macrophage population in wounds; (c ) M1 macrophage concentration in wounds; (d) M2 macrophage concentration in wounds.

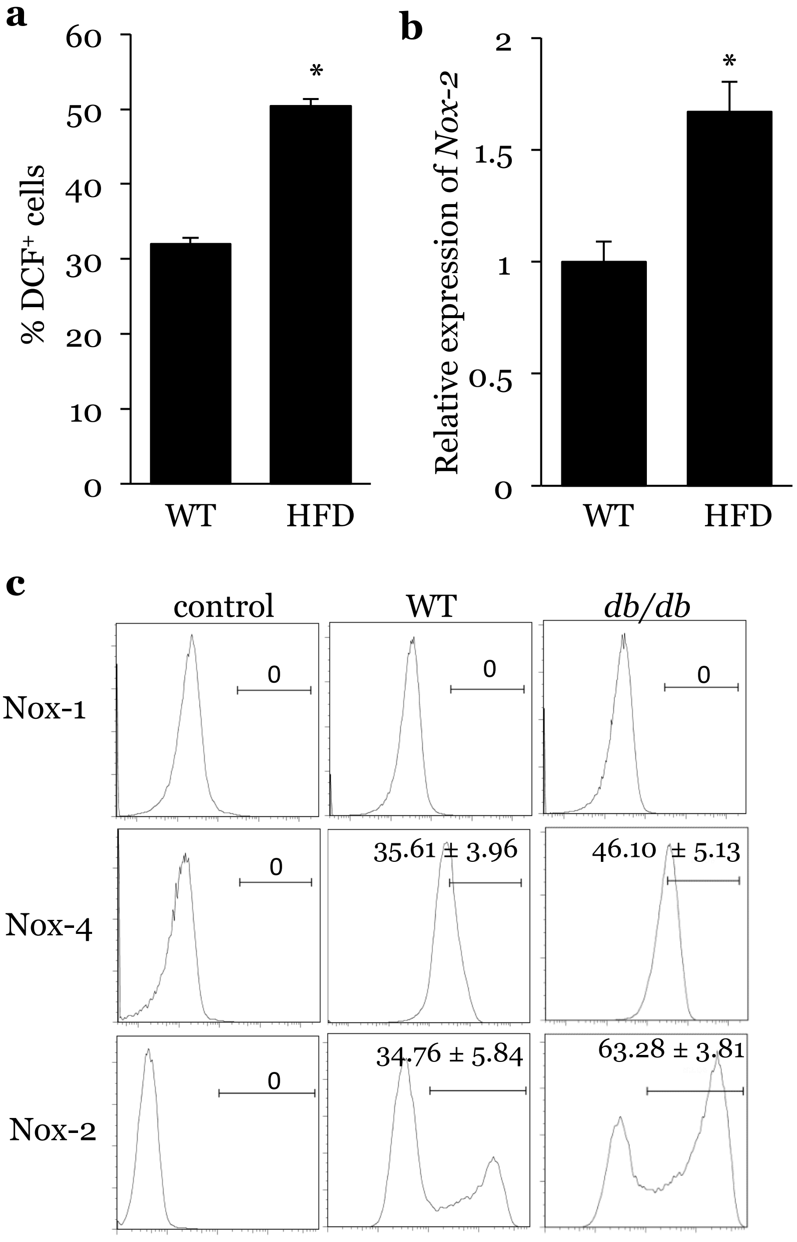


**Supplementary Figure 6. Oxidative stress and *Nox-2* expression in T2D HSCs**. Quantification of (a) DCF positive cells by flow cytometry (n=6, *, p<.05 vs WT); (b) gene expression by qRT-PCR (n=6, *, p<.05 vs WT); (c) Nox family by flow cytometry. Results are expressed as means ± SEM. Two-tailed unpaired student's t-test was used for **a**, **b** and **c**.

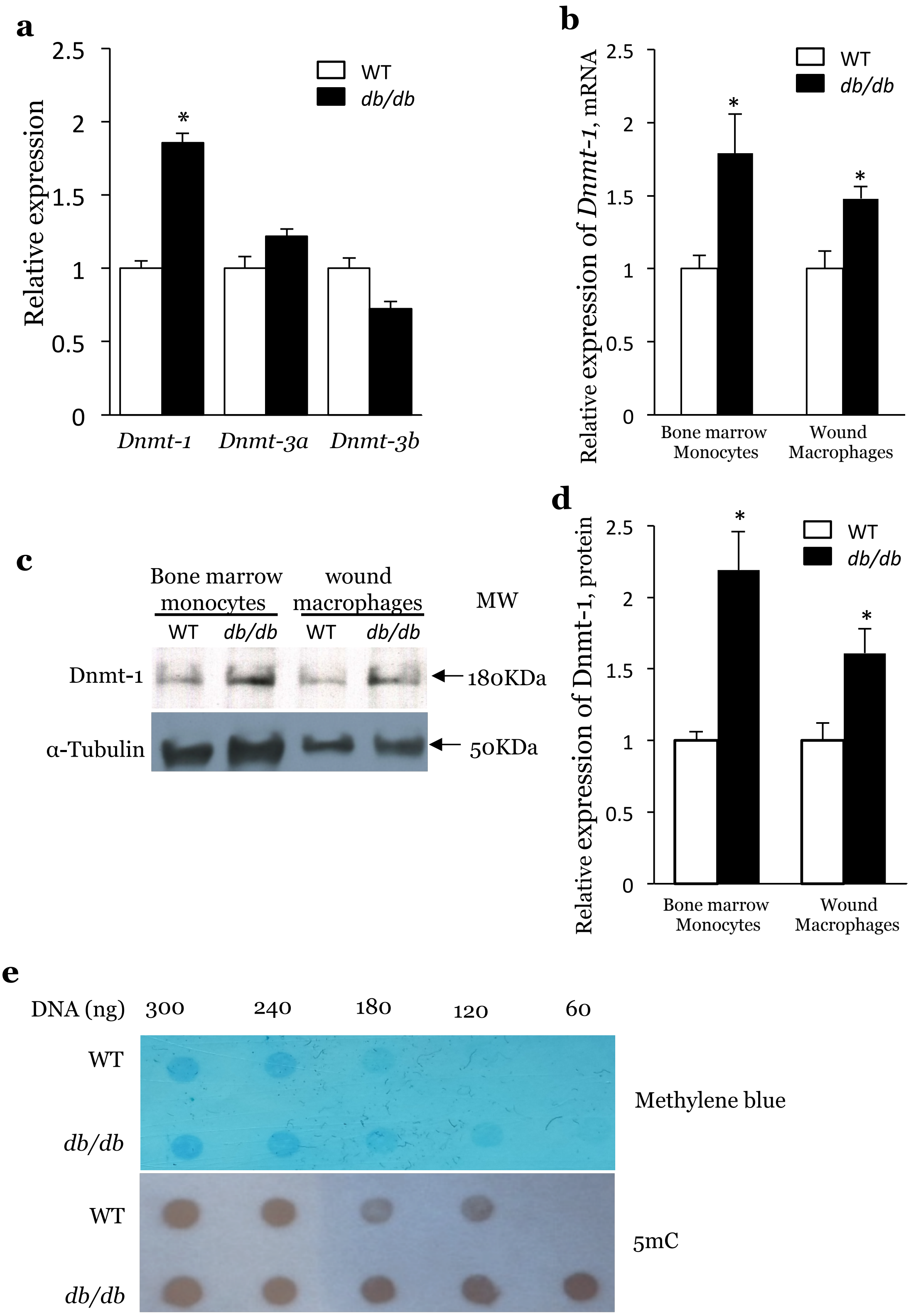


**Supplementary Figure 7. Dnmt-1 expression in *db/db* monocytes and macrophages.** (a) The expression of Dnmt family in HSCs from WT and *db/db* mice.(b-d) Quantification of Dnmt-1 expression in bone marrow monocytes and wound macrophages by qRT-PCR and western blot (n=6, *, p<.05 vs wt, #, p<.05 vs db/db). Results are expressed as means ± SEM. (d) Global DNA methylation analysis by dot blot. Two-tailed unpaired student's t-test was used for **a**, **b** and **d**.

**a**

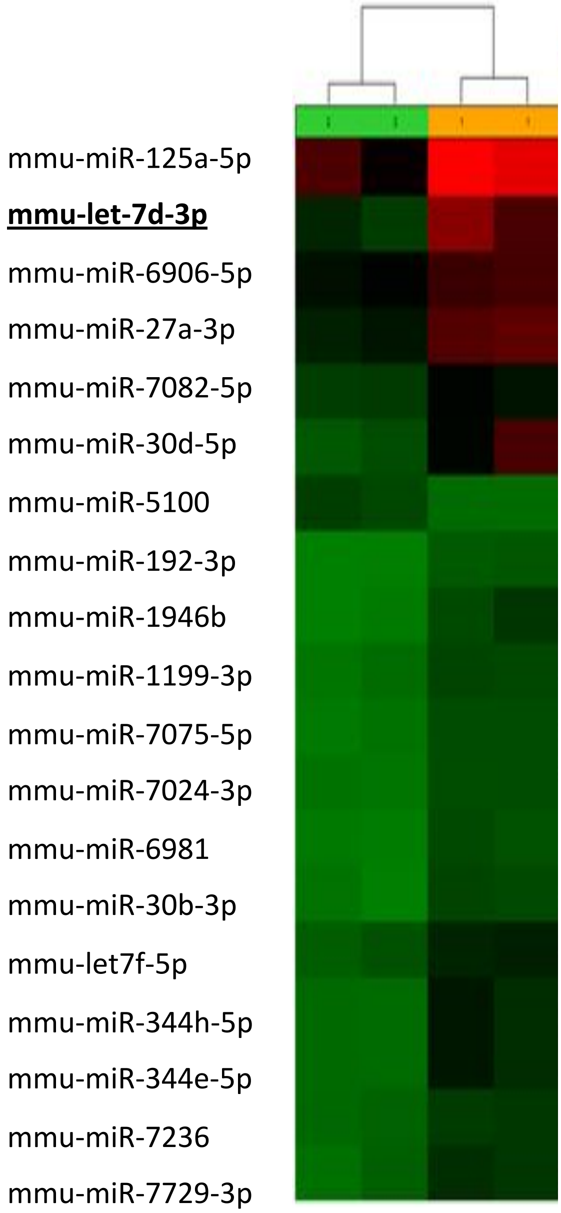


**b**

mmu-**let-7d-3p** sequence
CUAUACGACCUGCUGCCUUUCU

***Dnmt-1*** 3' UTR Sequence

**1 TGCTCTCACC CAGAGCCCCA CGTGCACTGA TGTTTTTAAC CCTTTGAGCC CCATCATTTG**
**61 AAGTCTTGTG CTCAGTGTCT GTGGCCATGG CTGACACTAA GCTGTTTGTA TGAGGTTTGT**
**121 TTTGTGACCA AGCTGTGTAG TACTTTGTGC ATTCTGAATT TTAAGGTTTT TTTTTTTGTT**
**181 TGGTTTGGTT TGGTTTGGTT TTTTTCTTAT CCTGTATTCT ATCAGATCTG CCACTGTGCA**
**241 GGTGGCAAGT GAGACTTGAT GTAGTTTTAT ATGTTGTAAT ATTTCTTCAA AATAAAGCGC**
**301 TTCTGTCAAG CACCC**

**Supplementary Figure 8. MicroRNA microarray expression data from WT and *db/db* HSCs.** (a)Heat map of microRNA microarray (b) Binding sequence of let-7d-3p in *Dnmt-1* 3’ UTR

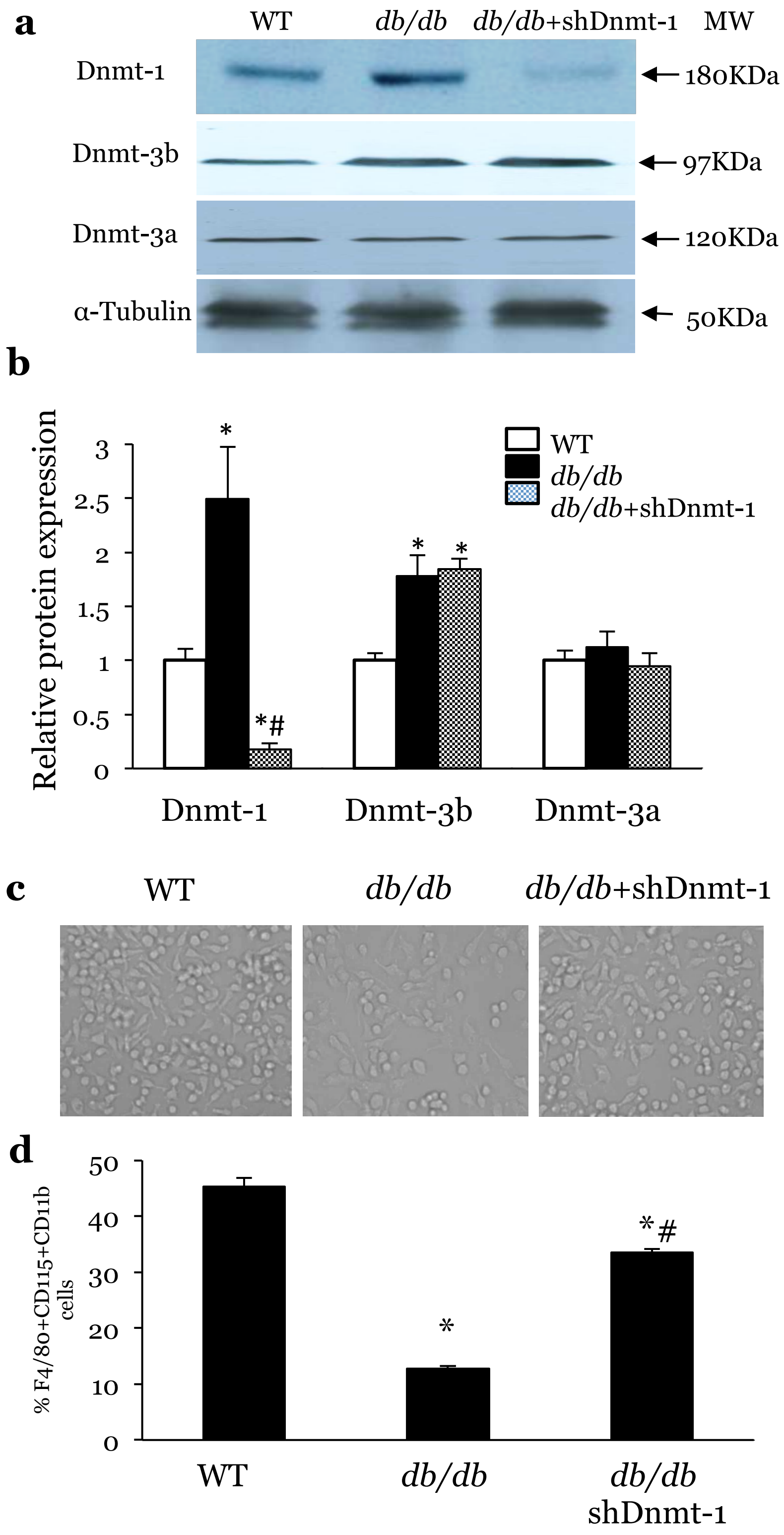


**Supplementary Figure 9. Dnmt-1 expression in *db/db* HSCS impairs their differentiation towards macrophages.** (a-b) Quantification of Dnmts protein expression by western blot (n=6, *, p<.05 vs wt, #, p<.05 vs *db/db*). (c ) *db/db* HSCs showed significantly decreased differentiation towards macrophages while (d) knockdown of Dnmt-1 significantly increased their differentiation towards macrophages ( n=6, *, p<.05 vs WT; #, p<.05 vs *db/db*). Results are expressed as means ± SEM. One way ANOVA was used for **b** and **d**.

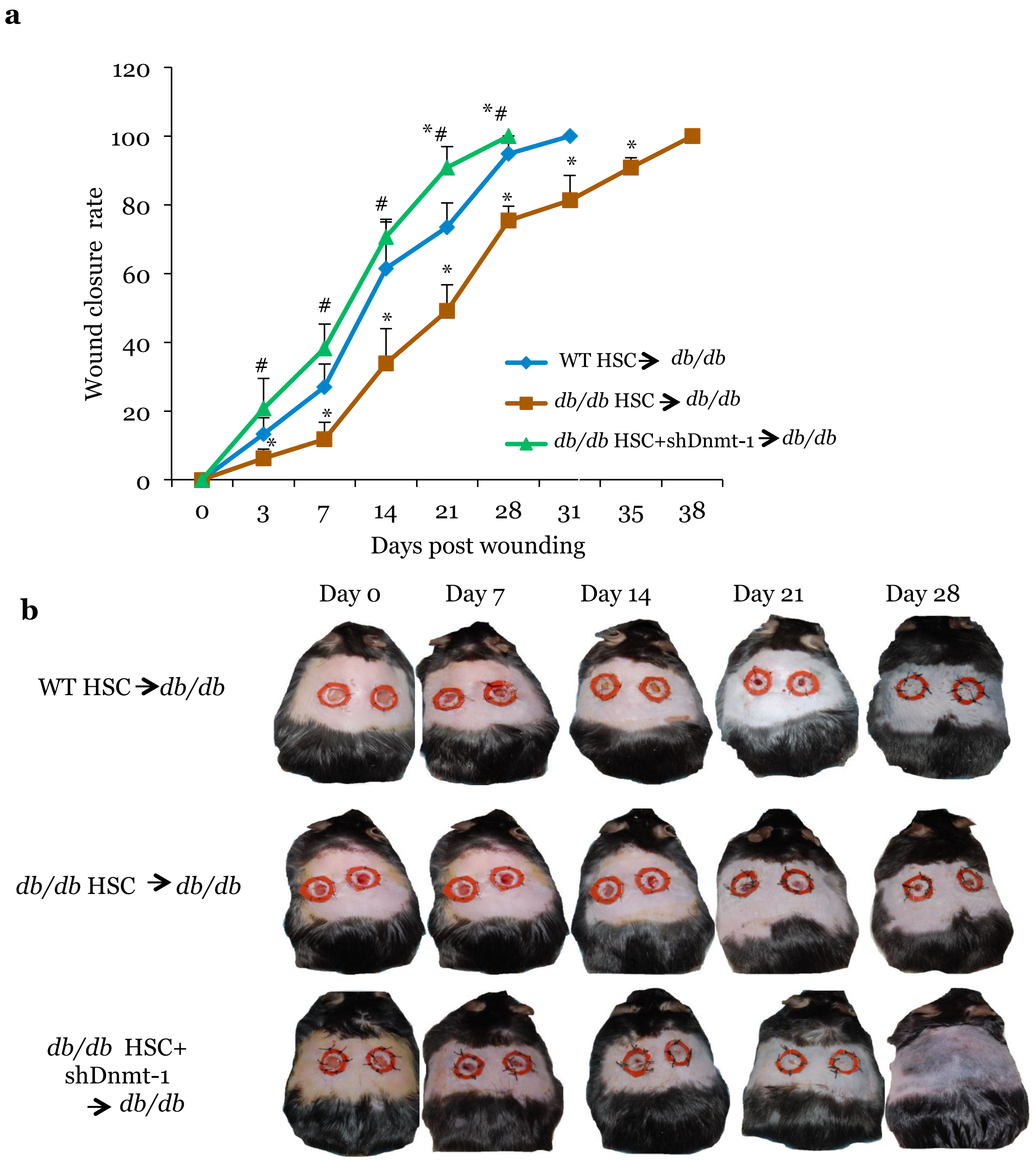


**Supplementary Figure 10. Dnmt-1 knockdown in T2D HSCs improves wound healing in T2D recipients**. (a) Wound closure rate measurement (n=8, *p<.05 vs WT HSC→ *db/db* #, p<.05 vs *db/db* HSC → db/db). (b) Representative images. Results are expressed as means ± SEM. One way ANOVA was used for **a**.

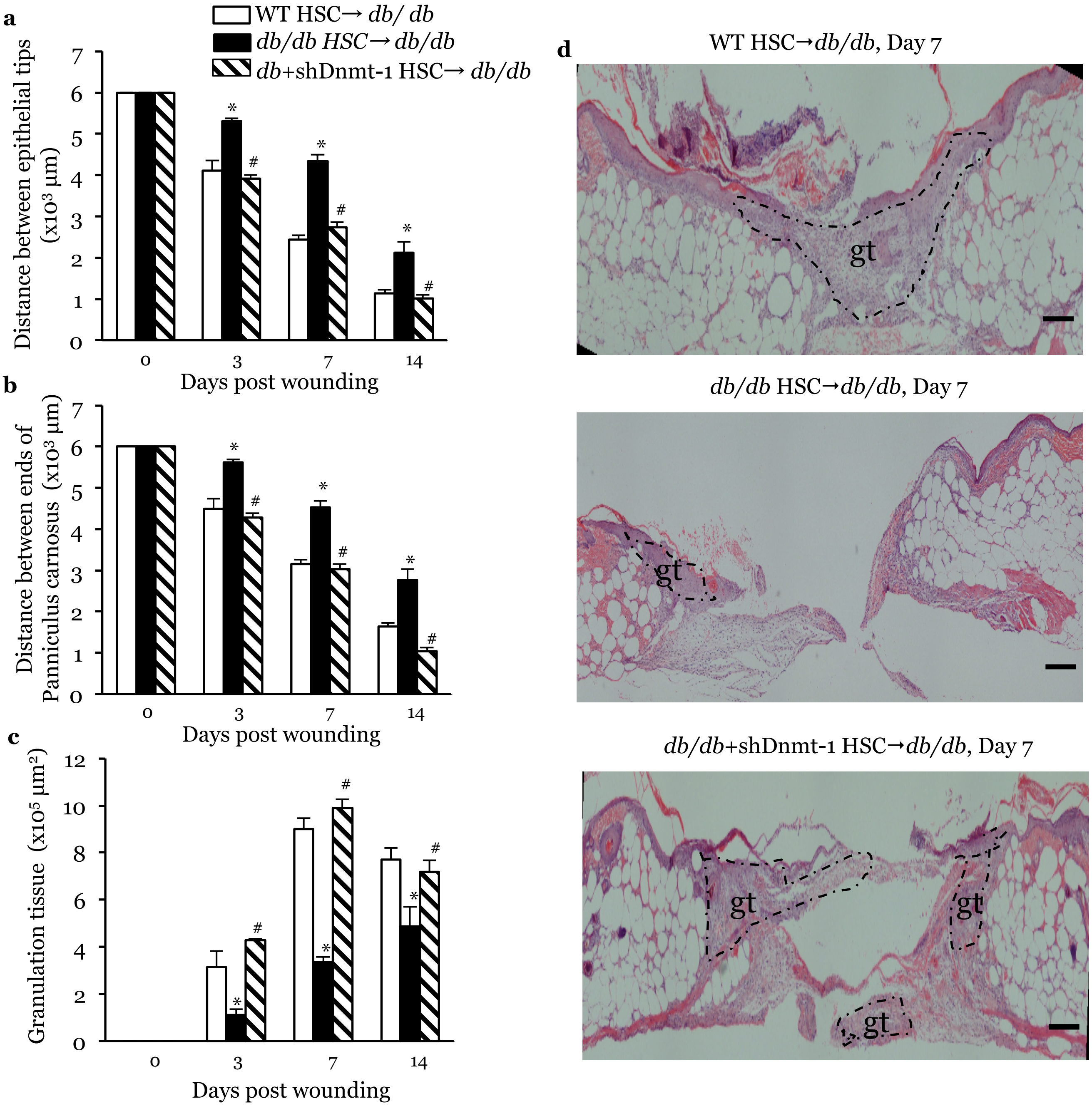


**Supplementary Figure 11. Morphometric quantification of wound closure.** (a-c) distance between epithelial tips/ends of panniculus carnosus and granulation tissue in wounds of *db/db* recipient mice. (n=4, *, p<.05 vs wt HSC→ *db/db*; #, p<.05 vs *db/db* HSC → *db/db*). Results are expressed as means ± SEM. (d) Representative H&E staining of wounds, magnification x40. Scale bar 100 μm. gt: granulation tissue. One way ANOVA was used for **a, b** and **c**.

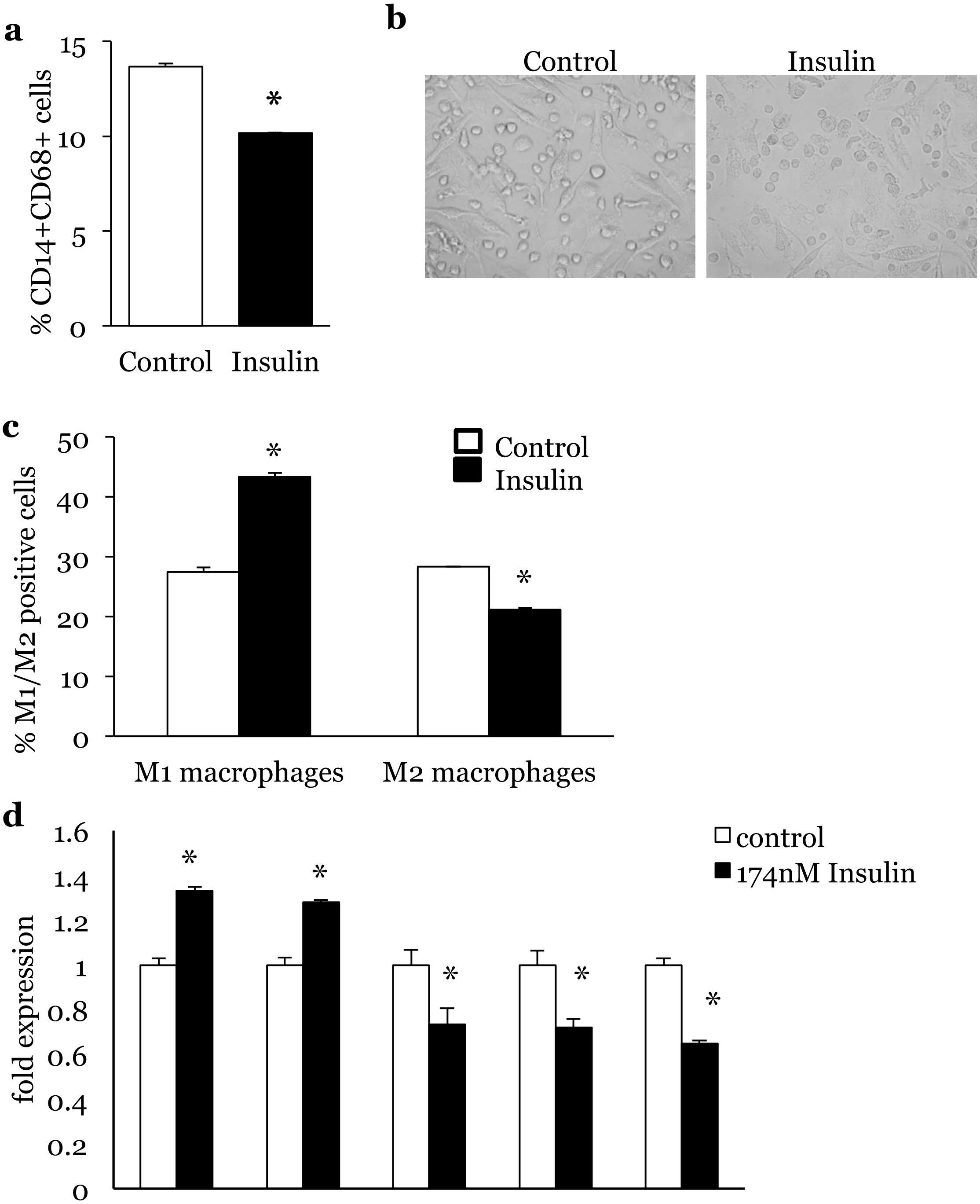


**Supplementary Figure 12. Hyperinsulinemia impairs human HSCs differentiation towards macrophages.** (a) Flow cytometry quantification of macrophage numbers (n=3, *p<.05 vs control). (b) Representative images. (c) Flow cytometry quantification of M1 and M2 macrophages (n=3, *p<.05 vs control. (d) Gene expression analysis (n=3, *, p<.05 vs control). Results are expressed as means ± SEM. Two-tailed unpaired student's t-test was used for **a**, **c** and **d**.

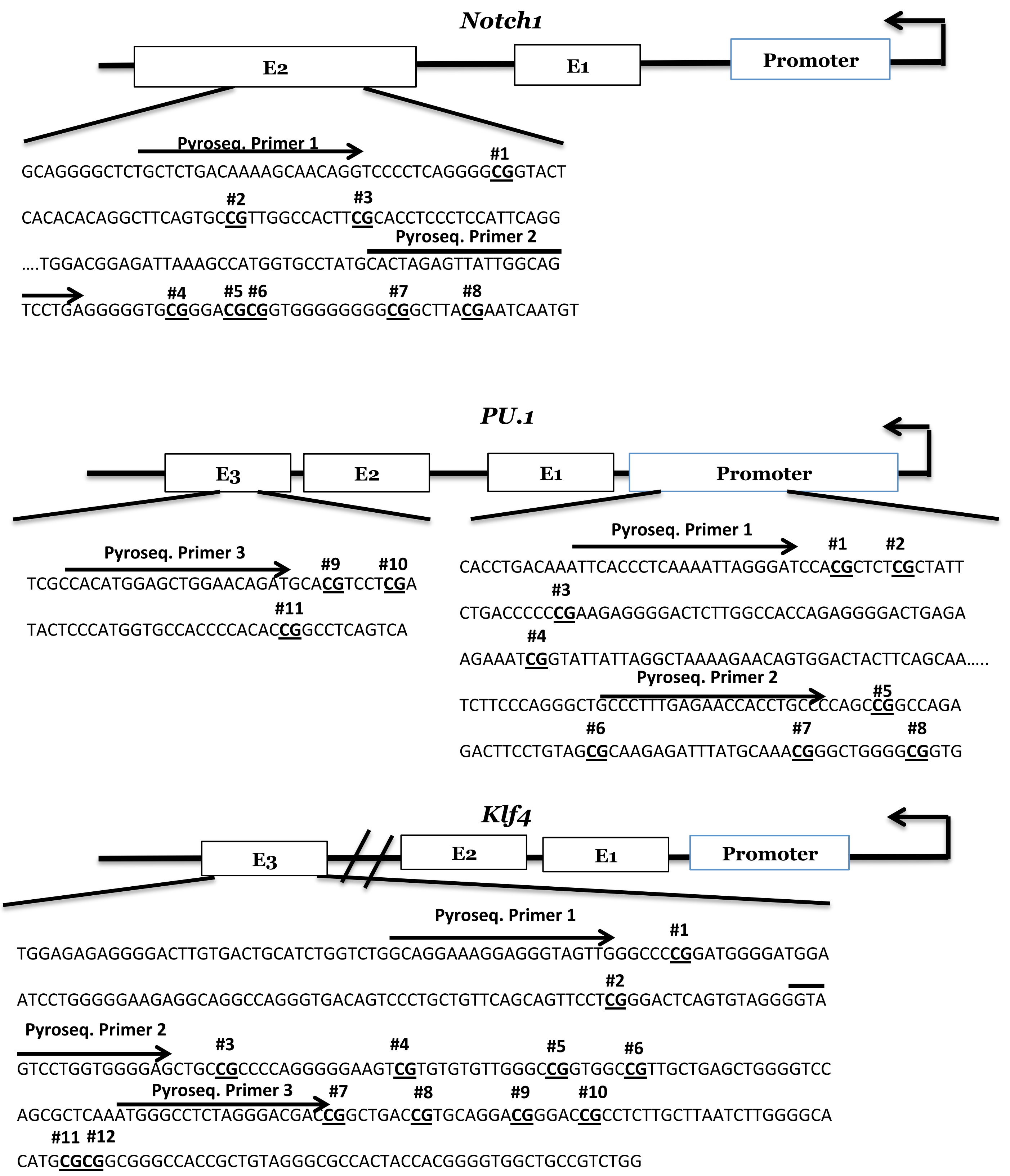


**Supplementary Figure 13. Schematic of the *Notch1*, *PU.1* and *Klf4* genes showing the sequence and location of the primers and the CpG islands tested by pyrosequencing.**

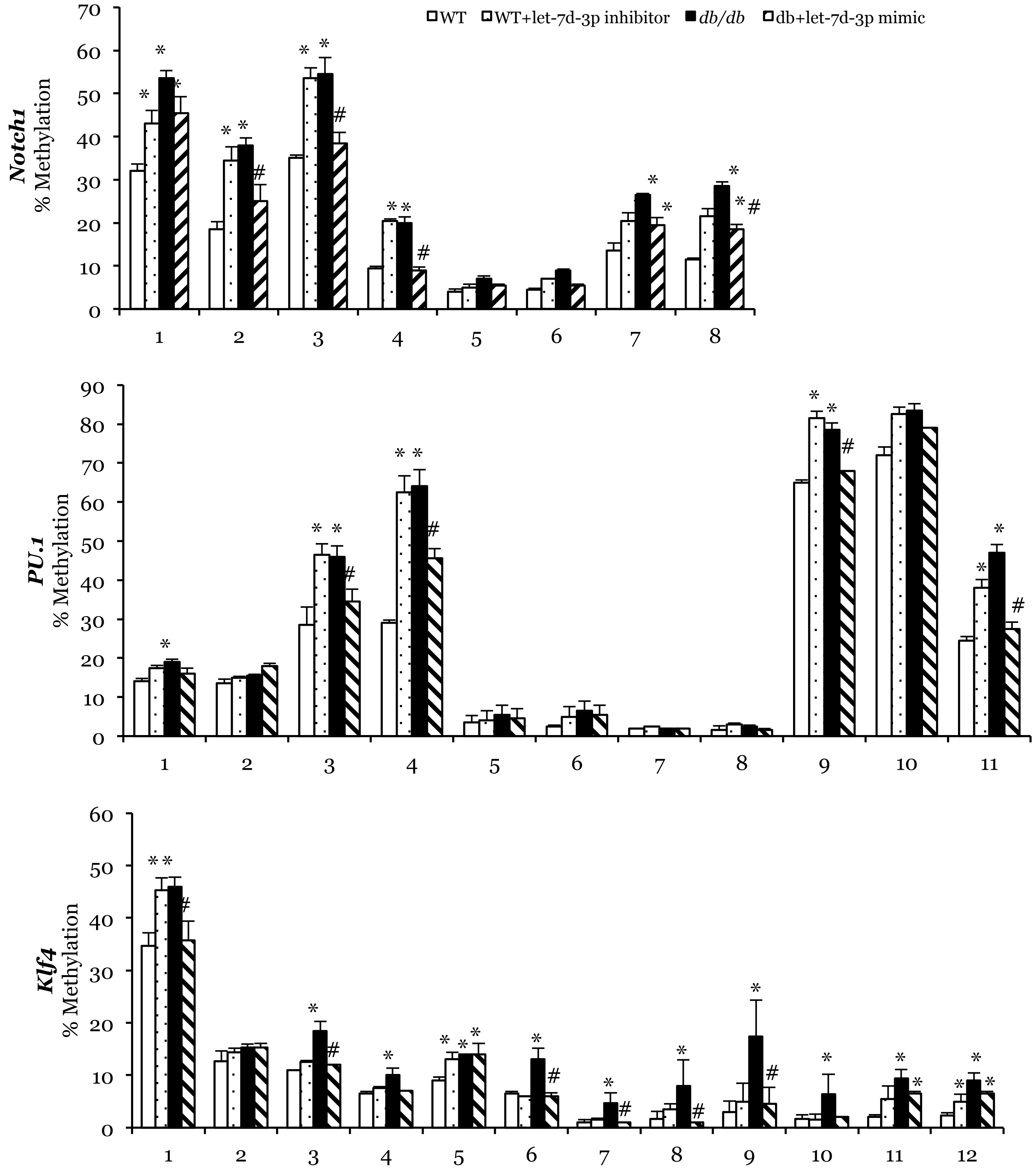


**Supplementary Figure 14. T2DM downregulates let-7d-3p in *db/db* HSCs, which leads to the increased methylation of *Notch1*, *PU.1* and *Klf4*.** (n=6, *, P<.05 vs WT; #, P<.05 vs *db/db*). Results are expressed as means ± SEM. One way ANOVA was used for analysis.

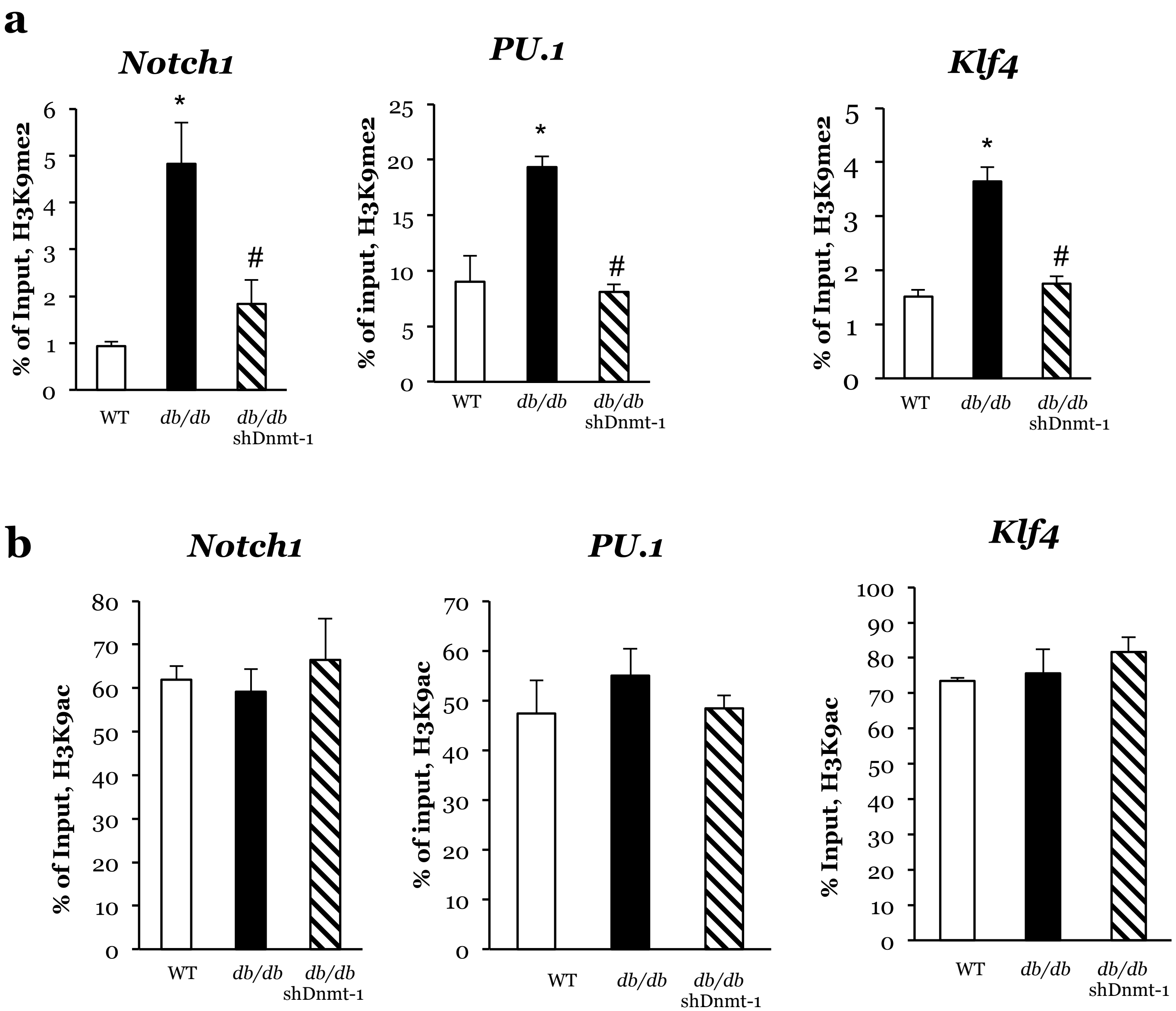


**Supplementary Figure 15. Dnmt-1-dependent regulation of histone modifications in *Notch1*, *PU.1* and *Klf4*.** ChIP-PCR analysis of histone modifications normalized to H3 (n=6. *, $p<.05$ vs WT; #, $p<.05$ vs *db/db*). Results are expressed as means ± SEM. One way ANOVA was used for **a** and **b**.

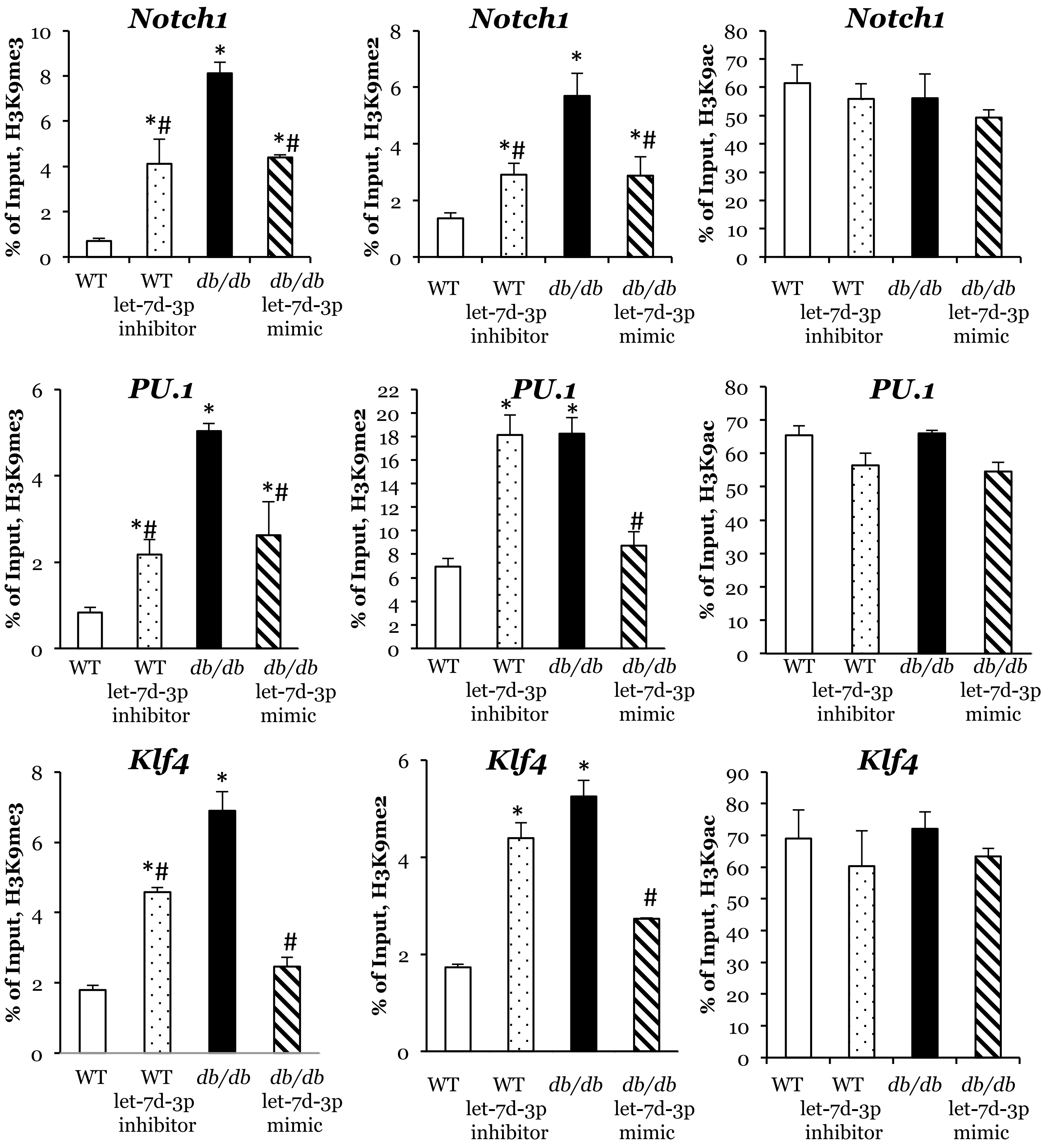


**Supplementary Figure 16. Let-7d-3p-dependent histone modification of *Notch1*, *PU.1* and *Klf4*.** ChIP-PCR analysis of histone modifications normalized to H3 (n=6. *, p<.05 vs WT; #, p<.05 vs *db/db*). Results are expressed as means ± SEM. One way ANOVA was used for analysis.

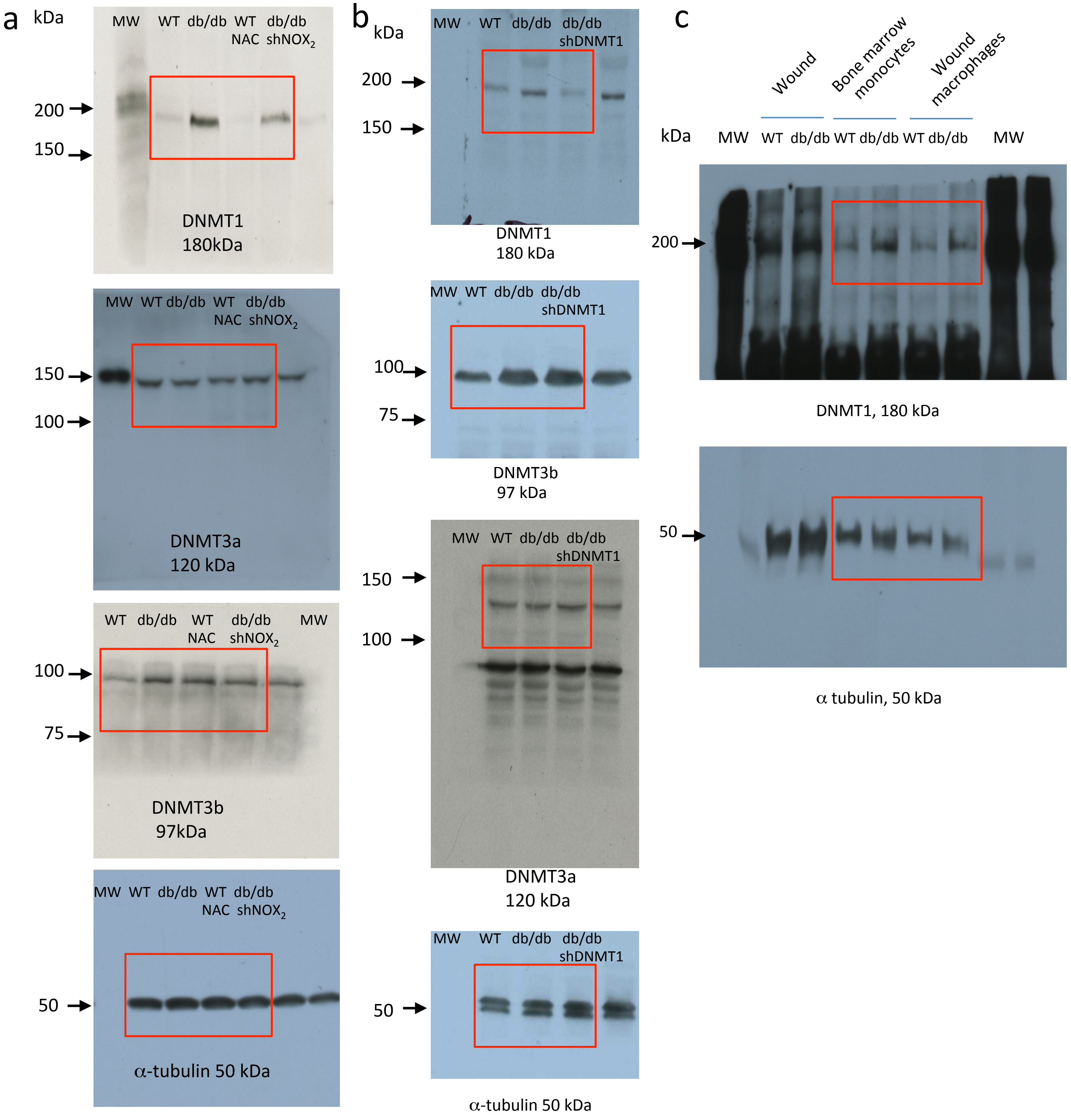


**Supplementary Figure 17. Uncropped original images of Western Blots.** Immunoblot data corresponding to (a) Fig. 4h (left column); (b) Supplementary Figure 9a (middle column) and (c) Supplementary Figure 7c (right column).

Supplementary Table 1. Mouse primers used for quantitative real time PCR

| Genes | Forward Primer (5'-3') | Reverse Primer (5'-3') |
|---|---|---|
| *Dnmt-1* | CACCTAGACGACCCTAACCTG | AGGTGGAGTCGTAGATGGACA |
| *Dnmt-3a* | AGCGTCACACAGAAGCATATCCAGGAG | GGCCAGTACCCTCATAAAGTCCCTTGC |
| *Dnmt-3b* | ATGGAATTGCAACGGGGTACTTGGTGC | CTGGCCTTCATGCTTAACAGTTCCCAC |
| *Notch1* | ATGCTGCTGTTGTGCTCCT | CAGTCTCATAGCTGCCCTCAC |
| *PU.1* | AACATAACTGGGCAAACCTGTAATA | GGTCCTTATCAACTTGGGTTATTCT |
| *Klf4* | CGGATCCGATGAGGCAGCCACCTGGC | CGACGCGTGCAAAGTGCCTCTTCATGTGTAAG |

Supplementary Table 2. Human Primers used for quantitative real time PCR

| Genes | Forward Primer (5'-3') | Reverse Primer (5'-3') |
|---|---|---|
| *NOX-2* | GTCACACCCTTCGCATCCATTCTCAAGTCAGT | CTGAGACTCATCCCAGCCAGTGAGGTAG |
| *DNMT-1* | TACCTGGACGACCCTGACCTC | CGTTGGCATCAAAGATGGACA |
| *NOTCH1* | GGCTAACAAAGATATGCAGAACAAC | GGTCCATATGATCCGTGATGT |
| *PU.1* | ATCAGAAGACCTGGTGCCCTAT | CAGTAATGGTCGCTATGGCTCT |
| *KLF4* | GGCGGGCTGATGGGCAAGTT | TGCCGTCAGGGCTGCCTTTG |
| *18 s* | GTAACCCGTTGAACCCCATT | CCATCCAATCGGTAGTAGCG |

Supplementary Table 3. List of primers used for pyrosequencing

| Genes | Forward Primer (5'-3') | Reverse Primer (5'-3') | Sequencing primers | Amplicon size (bp) |
|---|---|---|---|---|
| *Notch1* #1 | AAGGTAGGGGTTTTGTTTTGATAAAA | ACCAAACCTAACCTCTCTCT | TTGTTTTGATAAAAGTAATAGGT | 171 |
| *Notch1* #2 | TGGTGGAAGGAGATTAAAGTTATGGTG | TCCCCACTAATTTTTAACTACAATACTATA | TAGAGTTATTGGTAGTTTT | 137 |
| *PU.1* #1 | GTTAAGTTTGGTAGTTTTGGGATTAAAG | AAACCTTACTAAAATAATCCACTATTCT | AAATTTATTTTTAAAATTAGGG | 179 |
| *PU.1* #2 | AGGGTTGTTTTTTGAGAATTATTTGT | ATAATCCCCTTAAAATAACATCAC | GTTGTTTTTTGAGAATTATTTGTT | 107 |
| *PU.1* #3 | TTGGTTTTTTTATAGATTATTATTGGGATT | AACCAACAACACACACCTAATA | ATGGAGTTGGAATAATA | 222 |
| *Klf4* #1 | GGGGATTTGTGATTGTATTTGG | ATCCCTAAAAACCCATTTAAAC | GTAGGAAAGGAGGGTA | 238 |
| *Klf4* #2 | GGGATGGAATTTTGGGGGAAGAG | AAACCCATTTAAACCCTAAACCCCAACTCA | GTAGTTTTGGTGGGGA | 173 |
| *Klf4* #3 | TTGGGGTTTAGAGTTTAAATGGGT | ACAACAACCCTTCCATCATCAATATTAACA | ATGGGTTTTTAGGGAT | 176 |

Supplementary Table 4. List of primers used for ChIP-qPCR

| Genes | Forward Primer(5'-3') | Reverse Primer(5'-3') |
|---|---|---|
| *Notch1* | CCAGAGGCATTTAAATAAACAG AGA | TGGAAATTCTTGTCCTCTTATTTTG |
| *PU.1* | GGTAATGGCTAAGCTGAAATCT TCC | TTTGGTTTCATGTTGTCTCCTGATG |
| *Klf4* | TGCCTTGCTGATTGTCTATTTTT AT | CTTTAGAAACAAAACCTCAAACCAA |
